# Updates on the Prognosis of Giant Cell Arteritis: A Systematic Review

**DOI:** 10.7759/cureus.50299

**Published:** 2023-12-10

**Authors:** Abdelrahman M Abukanna, Yousef F Alanazi, Fahad Wadi S Alanazi, Rayan A Alanazi, Saif S Alanazi, Jarah T ‏Alenezi, Hussam K Alenezi, Abdulaziz Wadi S Alanazi

**Affiliations:** 1 Internal Medicine, Northern Border University, Arar, SAU; 2 Medicine, Northern Border University, Arar, SAU; 3 Medicine, Arabian Gulf University, Manama, BHR

**Keywords:** giant cell arteritis prognosis, systematic review, giant cell arteritis updates, prognosis, giant cell arteritis

## Abstract

Giant cell arteritis (GCA) is considered the most common type of vasculitis, especially in people aged 50 years or older, and imaging studies have helped predict its systemic nature. We conducted this review to highlight the results of the recently published articles considering the prognosis of giant cell arteritis (GCA). We searched for the relevant literature in SCOPUS, PubMed, Web of Science, and Science Direct and were included. We used Rayyan (Rayyan Systems, Cambridge, Massachusetts) throughout this systematic approach. The search resulted in twelve studies with 2600 patients with GCA diagnosis; most of them, 1853 (71.3%), were females. This systematic review found that most of the GCA patients experienced at least one relapse episode, primarily in patients younger than 75 years, with dependency on glucocorticoids, female sex, and involvement of large vessel vasculitis. We also found that stroke in GCA patients was associated with a bad prognosis. Therefore, we think more prospective studies are needed to enhance particular patient outcomes, and new therapeutic approaches using accessible biotherapies like tocilizumab and other similar medications are required.

## Introduction and background

Giant cell arteritis (GCA) is a type of vasculitis affecting large vessels, and it affects people over 50 years and is more common in people in their 70s and 80s [1,2]. It involves the major arteries, like branches of the external carotid arteries and the ophthalmic and vertebral arteries, and the etiology of inflammation and vascular remodeling [3,4] is not fully known [3,4]. It is distinguished by the co-occurrence of systemic symptoms like weight loss, anorexia, fatigue, fever, polymyalgia rheumatica (PMR), and aortitis with ischemia symptoms including headache, visual abnormalities, scalp discomfort, jaw claudication, and stroke. Aortic involvement can be worsened by aneurysm and aortic dissection, the prevalence of which is thought to be roughly 19 per 1000 patients per year [5].

If the diagnosis is made later than necessary, there could be catastrophic consequences, such as sight loss, stroke, and aortic aneurysm. Many GCA patients first see their family doctor or neighborhood emergency room. Headache is the primary presenting symptom of GCA (76%); however, other causes of headache are substantially more prevalent in these circumstances due to their relative frequency [6].

GCA is relatively uncommon in a non-specialist situation, with a frequency of 7-29 per 100,000 people over 50 in Europe. People of northern European heritage are more likely to have it [7]. With a mean onset of 70, the frequency rises with age and is highly uncommon in individuals under 50. It is more prevalent in women than in men (two to six times more likely), as is the case with many other autoimmune illnesses [8].

Macrophages (which may merge to produce the recognizable giant cells) and CD4+ T lymphocytes are present in the blood vessel wall damaged by inflammation, such as the carotid artery and its branches. This causes the intima to thicken, which reduces blood flow and causes ischemia, which is the primary source of pain in the areas that the damaged vessel supplies (for instance, temporal artery involvement can induce headache). There are also systemic symptoms due to cytokine release [9].

Ocular symptoms occur in almost two-thirds of patients with giant cell arteritis (GCA), and up to 30% of these patients develop permanent visual loss. In GCA, there is a preference for branches of posterior ciliary arteries. Anterior arteritic optic neuropathy is the most typical mechanism of visual loss in GCA, and it is caused by vasculitic involvement of short posterior ciliary arteries. Central retinal artery occlusion is the second most common cause leading to visual loss in patients with GCA. The diagnosis is GCA when the patient is over 50 years old and has both anterior ischemic optic neuropathy and a central retinal artery occlusion [10].

Permanent sight loss is the most dreaded long-term result of GCA, so it is critical to identify the symptoms quickly and treat people who have a suspected GCA. Aortic aneurysm development has been estimated to occur in 10% to 20% of patients. Although less often, aortic dissection and stroke have also been mentioned [11]. This systematic review investigates the recently published articles on the prognosis of patients with GCA.

## Review

Methodology

This systematic review was conducted according to the widely known standards of Preferred Reporting Items for Systematic Reviews and Meta-Analyses (PRISMA) [12].

We conducted this systematic review in August 2023. The review interpreted the collected data to improve the insights into the recent updates related to giant cell arteritis and to identify the areas that can be expanded by further research. 

Search strategy

Two of the authors did a detailed search of the major databases, which included SCOPUS, PubMed, Web of Science, and Science Direct, to find the relevant literature. We restricted the search to English-language publications and considered the unique database's requirements. The following keywords were converted into PubMed Mesh terms and used to find the relevant studies; "giant cell arteritis", "aortitis", "prognosis", "prognostic factors", and "outcomes." The Boolean operators "OR" and "AND" were matched to the required keywords. Publications with full English text, available free articles, and human trials were among the search results.

We considered the following criteria for inclusion in this review: study designs that investigated the recently published articles on the prognosis of patients with GCA; studies that included and discussed the fate of the disease among the patients; recently conducted studies between 2013 and 2023; full-text articles; and freely accessible articles.

We defined the exclusion criteria to ensure that the selected studies for the review were appropriate; they include Non-English language studies, repeated studies, case reports, research reviews, and articles with a primary objective other than updates on giant cell arteritis, as well as any unpublished research, books, grey literature, or comments to editors.

Data extraction

We used Rayyan (Rayyan Systems, Cambridge, Massachusetts) to examine the search strategy's output for duplicate information [13]. Using the previously mentioned inclusion/exclusion criteria, two authors (FW and YF) modified the combined search results to assess the relevance of the titles and abstracts to the review's objective. They thoroughly evaluated full-text papers that satisfied the requirements for inclusion and double-checked by two other authors (RA and SS). We discussed the methods for resolving conflicts and agreed to the conversation about the issues between all authors. For the final required data, we kept the complete texts of all pertinent studies that met the inclusion criteria. The authorized studies were uploaded in a data extraction form that had been prepared previously. The data that have been extracted included the study titles, study year, authors, country, objectives of the study, the participants and their gender, and the primary outcomes. The risk of bias was assessed in a separate sheet. We made summary tables utilizing data from relevant research to provide a qualitative analysis of the findings and study components. Once the data for the systematic review were retrieved, we chose the most efficient way to use the data from the included study articles. We identified the objectives related to the updates on the prognosis of giant cell arteritis.

Risk of bias assessment

We used the Risk Of Bias In Non-randomised Studies - of Interventions (ROBINS-I) risk of bias assessment method for non-randomized treatment trials to assess the quality of the included studies [14]. We considered seven parameters, including confounding, selection of participants for the study, intervention classification, deviations from intended interventions, assessment of outcomes, missing data, and the reported result selection.

Results

**Figure 1:**
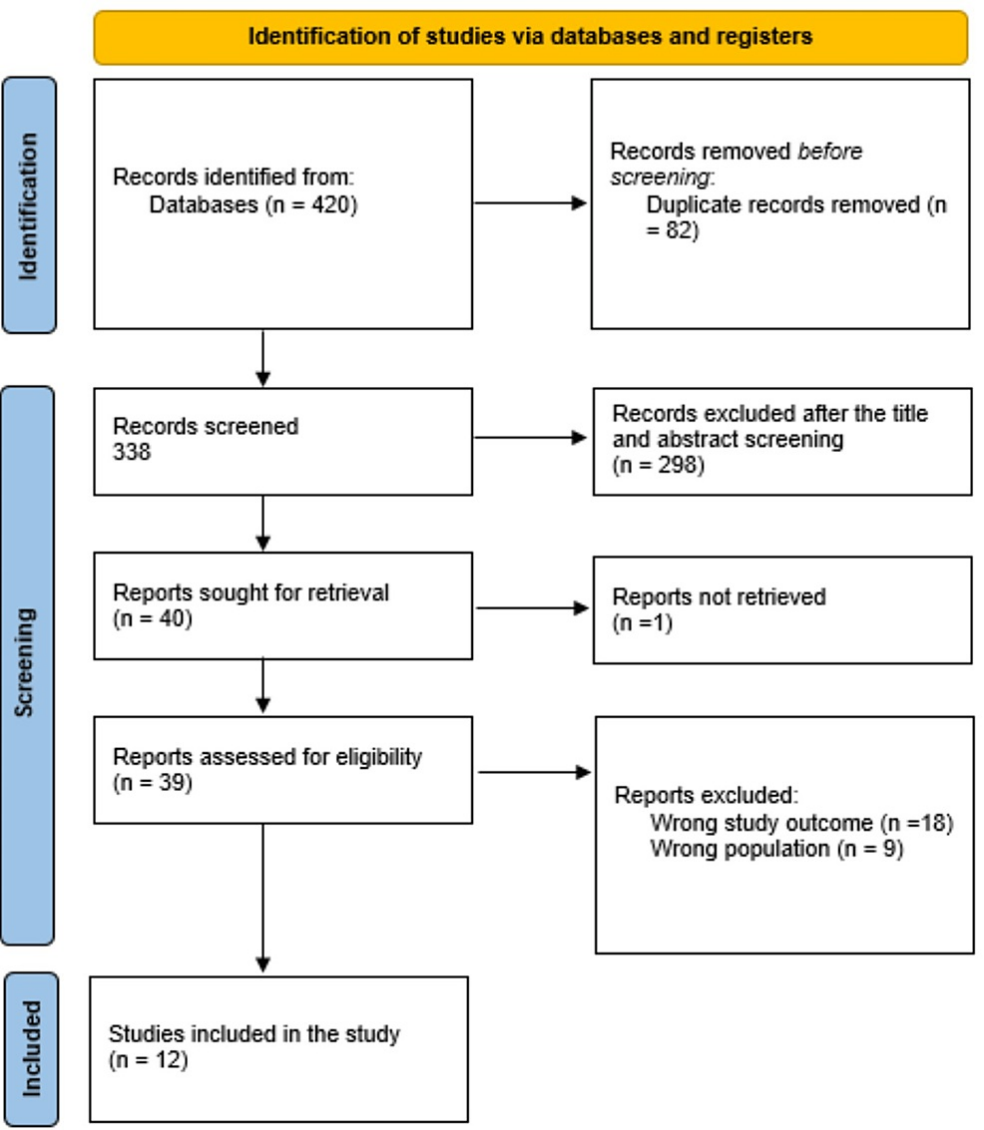
The study selection process summarized by a PRISMA flowchart PRISMA: Preferred Reporting Items for Systematic Reviews and Meta-Analyses

The systematic search resulted in 420 study articles, and 82 duplicates were deleted. The screening on title and abstract was conducted on 338 studies, and the reviewers excluded 298 studies. Then, 40 reports were sought for retrieval, and only one article was retrieved. Finally, 39 studies were screened for full-text assessment; 18 were excluded for wrong study outcomes, and nine were excluded because of the wrong participant type. The reviewers remained with twelve study articles included in the systematic review. A presentation of the summary of the study selection process is shown in Figure 1. 

Characteristics of the included studies

Table 1 presents the sociodemographic characteristics of the included study articles. Our results included nine studies with 2600 patients diagnosed with GCA, most of which 1853 (71.3%) were females. Seven studies were conducted in France [15-21], three in the United States of America (USA) [22-24], one in Austria [25], and one in Denmark [26]. Nine of the included studies were retrospective [15-17, 19-21, 23, 25, 26], one was prospective [24], one was a case-control [18], and one was cross-sectional [22].

**Table 1:** Sociodemographic characteristics of the included studies

Study	Study design	Country	Participants	Mean age (years)	Males (%)
Genin et al., 2023 [15]	Retrospective	France	81	66 ± 8	62 (76.5)
Zulfiqar et al., 2022 [16]	Retrospective	France	67	75.9 ± 8.5	43 (64.2)
Parreau et al., 2022 [17]	Retrospective	France	19	67.1 ± 5.3	11 (57.9)
Dumont et al., 2020 [18]	Case-control	France	104	78	68 (65)
Dumont et al., 2020 [19]	Retrospective	France	326	62	238 (73)
Espitia et al., 2021 [20]	Retrospective	France	171	70 ± 9	127 (74%)
Liozon et al., 2019 [21]	Retrospective	France	865	74.3 ± 8.26	590 (68.2)
Chean et al., 2019 [22]	Cross-sectional	USA	318	73.7 ± 8.2	222 (69.8%)
Kermani et al., 2013 [23]	Retrospective	USA	204	76.0 ± 8.2	163 (80%)
Unizony et al., 2021 [24]	Prospective	USA	224	68.7 ± 8.1	169.0 (75.4)
Jud et al., 2021 [25]	Retrospective	Austria	144	74.7	111 (77.1)
Emamifar et al., 2021 [26]	Retrospective	Denmark	77	71.8 ± 8	49 (63.6%)

Table 2 presents the clinical characteristics of the study population. Most of the included patients experienced at least one relapse episode [15], mainly with patients younger than 75 years [16], dependency on glucocorticoids [19, 24], of female sex [24], and involvement of large vessel vasculitis [19]. Stroke in GCA patients was associated with a bad prognosis [17, 19]. Regarding the ophthalmic manifestations, Patients with GCA experience severe and frequent visual symptoms before and several years after the diagnosis. Early and effective treatment is crucial because GCA-related symptoms can be avoided [18, 21, 22]. Serious complications, including aortic aneurysms or dissection, might result from aortitis in GCA [15,20]. Risk of bias assessment showed high risk in two studies [17, 20], moderate risk in seven studies [15, 16, 18, 22, 24-26], and unclear risk in three studies [19, 21, 23].

**Table 2:** Clinical characteristics and outcomes of the included studies GCA - giant cell arteritis, CTA - computed tomography angiography, FDG - fludeoxyglucose, PET - positron emission tomography, CT - computed tomography, GC - cluco-corticoid, LVV - large vessel vasculitis, PBO - placebo, PDN - painful diabetic neuropathy, OR - odds ratio, CI - confidence interval, p - probability, AD - aortic dilatation, IMT - inflammatory myofibroblastic tumor, PMR - polymyalgia rheumatica, TAB - temporal artery biopsy, ROBINS-I - Risk Of Bias In Non-randomised Studies - of Interventions

Study	Aim of study	Main outcomes	ROBINS-I
Genin et al., 2023 [15]	To evaluate the GCA-associated aortitis relapses of patients based on the detection of aortitis on CTA and/or FDG-PET/CT.	62% of patients experienced at least one relapse during follow-up. An increased risk of relapse was linked to aortic wall thickening. Positivity of both CTA and FDG-PET/CT for GCA-related aortitis was associated with an increased risk of relapse. Aortic wall thickening on CTA was a risk factor of relapse compared with isolated aortic wall FDG uptake.	Moderate
Zulfiqar et al., 2022 [16]	To prove the latter through a single-center investigation carried out on a group of people with GCA.	Patients over 75 years old experience less aortitis than younger individuals. In younger participants, there also seems to be a trend towards more relapses, with older patients receiving a clinically significant shorter follow-up of one year.	Moderate
Parreau et al., 2022 [17]	To more easily diagnose GCA, the characteristics of GCA-related stroke were contrasted with those of GCA without stroke and atherosclerosis-related or embolic stroke.	In 3-4% of instances, stroke complicates the course of GCA by either showing vasculitis or delaying the onset of the disease. A stroke can very rarely happen during a late GCA relapse. GCA-related stroke has a bad prognosis and might be challenging to diagnose.	High
Dumont et al., 2020 [18]	To describe the traits and prognosis of patients with ophthalmologic involvement associated with GCA at diagnosis.	25% of patients had GCA-related ophthalmologic involvement, and 10% did not recover their eyesight. These findings support and urge the need for the rapid development of preventative measures for these frequently irreversible ophthalmologic problems.	Moderate
Dumont et al., 2020 [19]	To recognize traits and elements in GCA patients that are connected to relapse and GC dependency.	Relapses and dependency on glucocorticoids are frequent in GCA. A separate predictor of relapse and GC dependency was large-vessel involvement. GC dependency and relapse were independently predicted by large-vessel vasculitis (LVV). On the other hand, a prior stroke was preventive of recurrence and GC dependence.	Unclear
Espitia et al., 2021 [20]	To look into the risk factors for aortic problems in a cohort of GCA aortitis patients.	Serious complications, including aneurysms or dissection, might result from aortitis in GCA. Those with symptomatic aortitis at the time of diagnosis of GCA, primarily chest and abdominal discomfort, may represent a unique sub-group of aortitis with more aortic aneurysm or dissection during follow-up than those with non-symptomatic aortitis.	High
Liozon et al., 2019 [21]	To compare them to those of younger patients, the initial characteristics, course, and prognosis of GCA in individuals under the age of 85 were examined.	A GCA diagnosis can be made easily for the majority of patients under the age of 85, potentially reducing morbidity connected to vision. The extremely elderly population appears to be amenable to corticosteroid treatment at usual or high doses (pulse methyl-prednisolone), but this may come with increased toxicity, most notably infections.	Unclear
Chean et al., 2019 [22]	To describe GCA patients and compare the general and GCA-specific traits of those who are currently experiencing visual problems and with no visual symptoms	Patients with GCA continue to experience serious and frequent visual symptoms before and several years after diagnosis. Early, effective treatment is crucial because GCA-related symptoms can be avoided, and if visual symptoms are present, prompt care and referral to an ophthalmologist are required to stop development and, eventually, total blindness.	Moderate
Kermani et al., 2013 [23]	To analyze the impact of LV manifestations on survival and to assess the incidence trends and timing of LV manifestations in patients with GCA.	Aortic aneurysm/dissection is linked to lower survival. The frequency of aortic aneurysm/dissection increased five years after the diagnosis of GCA and persisted throughout the observation period, highlighting the need for ongoing monitoring of these patients. More mortality from cardiovascular causes, such as ischemic heart disease in patients with aortic aneurysm/dissection, occurred in patients with GCA and aortic symptoms.	Unclear
Unizony et al., 2021 [24]	To predict treatment failure in GCA patients getting tocilizumab in addition to glucocorticoids and in GCA patients taking only glucocorticoids	The two most potent risk factors for treatment failure in GCA have been discovered: prednisone monotherapy and female sex. In the PBO/PDN group, the risk of treatment failure was noticeably higher in women than in males (OR, 5.2; 95% CI, 1.6 to 17.2; p=0.007).	Moderate
Jud et al., 2021 [25]	To estimate AD frequency in a monocentric sample of GCA patients using CT and to find potential biomarkers linked to aortic dilatations.	Patients with GCA are more likely to experience thoracic aortic dilatation than abdominal ones, but there is no danger of aortic repair, rupture, or dissection afterward. Aortic dilation is linked to changes in T-cell subsets, the prevalence of polymyalgia rheumatica, and higher carotid IMT at disease onset.	Moderate
Emamifar et al., 2021 [26]	To assess how the inflammatory process and glucocorticoid therapy affect PMR/GCA body composition and aortic arterial stiffness.	Glucocorticoid therapy exhibited numerous prognostic effects in patients with PMR/GCA. A decrease in arterial stiffness was brought on by glucocorticoid treatment, and this decrease occurred regardless of TAB and 18F-FDG PET/CT outcomes. Anti-osteoporotic drugs are advantageous since they stop additional bone loss and have to be started at the same time as glucocorticoids.	Moderate

Discussion

This systematic review provides a practical update on the prognosis and outcomes of GCA. Serious problems, such as vision loss, can be avoided with early detection and treatment of GCA. The visual loss might develop and persist even after treatment. Before corticosteroid therapy was utilized to treat GCA, 30-60% of individuals had visual loss [27]. Within days to weeks of exhibiting symptoms, up to 60% of GCA patients could experience unilateral visual loss if ignored [28].

We reported that most of the included patients with GCA experienced at least one relapse episode [15], mainly with patients younger than 75 years [16], dependency on glucocorticoids [18, 23], of female sex [23], and involvement of large vessel vasculitis [19]. Regarding the ophthalmic manifestations, patients with GCA experience severe and frequent visual symptoms before and several years after diagnosis. Early and effective treatment is crucial because GCA-related symptoms can be avoided. Additionally, most of the subjects in this study (71.3%) were females [18, 21, 22].

Unizony et al. found that patients with GCA taking tocilizumab in combination with prednisone were less likely to experience treatment failure by six times than those taking only prednisone. They also found that the most potent risk factor for treatment failure was female sex in patients treated with prednisone only. In contrast, there is an increased risk for treatment failure with lower prednisone doses and worse patient-reported outcomes at the study baseline in patients taking tocilizumab [18].

Vision loss is a severe and dreaded consequence of GCA that is frequently irreversible. Between 10% and 30% of GCA patients in population-based cohorts exhibited visual signs [29-31]. Anterior ischemic optic neuropathy caused by GCA was expected to occur annually in 1.3 out of every 100,000 people under the age of 50 years [31]. Due to earlier diagnosis and treatment, the proportion of GCA patients who initially reported visual symptoms has decreased recently [30]. In a cohort study done by Alba et al., they found that 64% of patients had at least one relapse, and 36% developed two or more relapses. The symptoms indicating relapse include polymyalgia rheumatica (PMR), large vessel (LV) symptoms, and cranial symptoms, including ischemic complications and systemic disease. They noted that relapse occurred mainly within the first two years of treatment. Osteoporosis was found to be one of the complications in relapses of GCA [32]. 

In the study done in Japan by Sugihara et al., they found that large vessel lesions were detected by imaging studies in about half of the Japanese patients at the baseline investigations. Large vessel lesions, like aortic lesions, were associated with poor treatment outcomes in patients receiving conventional immunosuppressive medications with no biological agents. The intensity of initial treatment could be determined based on the presence or absence of large vessel lesions [33].

Once glucocorticoid therapy has begun, incident vision loss is uncommon; according to a retrospective study, it occurs about 1% of the time at five years [34]. Prospective clinical research found a higher prevalence of 14% at one year, which may be connected to the quick glucocorticoid taper [35]. Recurrent visual disturbances were observed by 5-10% of individuals in observational studies [36, 37].

The aorta and its branches are affected by GCA, and patients with LV signs may exhibit either constitutional or ischemia symptoms [38]. In 240 cases of fever or inflammation of unknown origin, a final diagnosis was made in 190 patients in a recent study using positron emission tomography (PET), and large-vessel vasculitis accounted for 21.1% of cases [39]. Recognizing LV involvement significantly affects how patients with GCA are evaluated and followed up. The sensitivity of physical examination to detect LV anomalies is between 14 and 50 percent [40].

The lack of information and confusion surrounding routine imaging in individuals with GCA are reflected in published guidelines and recommendations. The American College of Cardiology guidelines advise imaging the aorta and branches in all newly diagnosed GCA patients to look for signs of aortic injury [41, 42]. In studies examining patients with GCA by CT angiography, aortic structural damage was noted at the diagnosis in 15% to 23% of GCA patients [43]. The British Society of Rheumatology recommends considering imaging tests when diagnosing GCA in patients with LV symptoms [44].

In this study, we found that stroke in GCA patients was associated with a bad prognosis [17, 19]. Stroke may be a complication of vertebrobasilar illness caused by GCA [27]. The annual incidence of GCA-related stroke in persons under 50 years of age in a population-based investigation was calculated at 0.76 per 100,000 [45]. Seventy-nine percent of stroke cases in a group of 40 patients with GCA involved vertebrobasilar vessels. Most instances (73% at diagnosis and 27% within the first two weeks) occur shortly following diagnosis and starting treatment. This is an early manifestation of GCA [46].

The diagnosis, staging, and assessment of the disease extent of GCA may be made possible by more recent, non-invasive diagnostic testing, which includes sensitive modalities like positron emission tomography (PET), and there are currently investigations looking into the effectiveness of PET in assessing disease activity. Clarification is required regarding the importance and prognosis of LV anomalies seen on imaging. If there are "subsets" of individuals more likely to get ischemia or large-artery symptoms, they should be identified because they would benefit from more intensive treatment and surveillance. Understanding the significant contributors to vascular injury, especially aneurysms, is essential. Further research is required to determine if the management of aneurysms from GCA should follow the current guidelines based on the size of non-inflammatory aneurysms [23]. 

The guidelines for the treatment of giant cell arteritis are as follows: if the patient has an active disease with visual impairment or loss or cranial ischemia, the recommendation is to start high dose intravenous corticosteroids followed by high dose daily oral glucocorticoids with tocilizumab over glucocorticoid alone, and in some cases, we can consider the use of glucocorticoids with methotrexate or glucocorticoids alone. If there is clinical remission, giant cell (GC) can be tapered, and if there is no remission, an immunosuppressive agent should be added, like abatacept or methotrexate, instead of tocilizumab. If the patient has active disease without visual impairment, loss, or cranial ischemia, the recommendation is to start high-dose daily oral glucocorticoids with tocilizumab over glucocorticoid alone. Sometimes, we can consider using glucocorticoids with methotrexate or glucocorticoids alone. If there is clinical remission, GC can be tapered, and if there is no remission, an immunosuppressive agent should be added like abatacept or methotrexate instead of tocilizumab [47]

Further research is needed to find other efficient, targeted medicines similar to tocilizumab because it is beneficial in treating GCA. Assessing the ideal tocilizumab medication duration and the drug's long-term effects on significant illness outcomes is necessary [23].

The use of tocilizumab (TCZ) biosimilars will considerably reduce this agent's cost and change the strategy for the treatment of GCA, leading to earlier and more extensive use in the course of the disease. The improvements in understanding GCA's pathogenesis have opened up new ways and therapeutic opportunities. The discovery of new agents led to a new era in treating GCA. Of these new agents, the most promising at present are the IL12/IL23 inhibitors, the IL17 pathway inhibitors, abatacept modulators of T cells, the inhibitors of granulocyte-macrophage colony-stimulating factor (GM-CSF), and the endothelin family inhibitors (mainly ET1 inhibitors), especially with visual complications [48].

Study strengths

In this systematic review, we must consider many strengths that make it unique. The search strategy used different databases, saving time for relevant articles. The included articles were from a wide range of geographical regions and included diverse patient populations, making the findings' generalizability evident to the reader. The inclusion and exclusion criteria ensure that the selected studies adhere to the objectives and methodology. The overall result is an up-to-date overview of the prognosis of giant cell arteritis.

Study limitations

This systematic review has some limitations. These include the variability in the methodologies of selected studies, different patient populations, different geographical areas, and different outcomes in the selected studies. There was a potential for publication bias and reporting of the outcomes in the selected studies.

## Conclusions

This systematic review found that most of the GCA patients experienced at least one relapse episode, mostly with patients younger than 75 years, dependency on glucocorticoids, female sex, and involvement with large vessel vasculitis. It is also found that stroke in GCA patients is associated with a bad prognosis and difficult diagnosis but is preventive for the recurrence of GCA and glucocorticoid dependence.

An important finding is that ophthalmic complications can occur before the diagnosis of GCA, and it needs early treatment with glucocorticoids to prevent total visual loss and improve the prognosis. Still, it is essential to avoid the use of prednisolone as monotherapy, especially in females, because it is found to increase treatment failure. More prospective studies and trials are needed to enhance particular patient outcomes, and new therapeutic approaches using accessible biotherapies, like tocilizumab and other similar medications, are needed. 
